# The Identification of Core Gene Expression Signature in Hepatocellular Carcinoma

**DOI:** 10.1155/2018/3478305

**Published:** 2018-05-27

**Authors:** Ning Li, Ling Li, Yongshun Chen

**Affiliations:** ^1^Department of Cardiology, Renmin Hospital of Wuhan University, Cardiovascular Research Institute, Wuhan University and Hubei Key Laboratory of Cardiology, Wuhan 430060, China; ^2^Institute of Hepatobiliary Diseases of Wuhan University, Zhongnan Hospital of Wuhan University and Hubei Key Laboratory of Medical Technology on Transplantation, Wuhan, Hubei 430071, China; ^3^Department of Clinical Oncology, Renmin Hospital of Wuhan University, Wuhan 430060, China

## Abstract

Hepatocellular carcinoma (HCC) is one of the most common malignancies, which causes serious financial burden worldwide. This study aims to investigate the potential mechanisms contributing to HCC and identify core biomarkers. The HCC gene expression profile GSE41804 was picked out to analyze the differentially expressed genes (DEGs). Gene ontology (GO) and the Kyoto Encyclopedia of Genes and Genomes (KEGG) analyses were carried out using DAVID. We constructed a protein-protein interaction (PPI) network to visualize interactions of the DEGs. The survival analysis of these hub genes was conducted to evaluate their potential effects on HCC. In this analysis, 503 DEGs were captured (360 downregulated genes and 143 upregulated genes). Meanwhile, 15 hub genes were identified. GO analysis showed that the DEGs were mainly enriched in oxidative stress, cell cycle, and extracellular structure. KEGG analysis suggested the DEGs were enriched in the absorption, metabolism, and cell cycle pathway. PPI network disclosed that the top3 modules were mainly enriched in cell cycle, oxidative stress, and liver detoxification. In conclusion, our analysis uncovered that the alterations of oxidative stress and cell cycle are two major signatures of HCC. TOP2A, CCNB1, and KIF4A might promote the development of HCC, especially in proliferation and differentiation, which could be novel biomarkers and targets for diagnosis and treatment of HCC.

## 1. Introduction

Primary liver cancer, the sixth most common cancer overall, is causing the second largest number of cancer death all over the world. Hepatocellular carcinoma (HCC) is a predominant primary liver cancer, accounting for approximately 90% of all types of primary hepatic malignancy and triggering a major international public health problem [1]. The pathogenesis of HCC is a multistep process implicated with the progressive accumulation of gene alterations that pinpoint various cellular and molecular events including oxidative stress, endoplasmic reticulum stress, and abnormal cell cycle [2]. Take oxidative stress, for example, the increased production of reactive nitrogen species (RNS) or reactive oxygen species (ROS), in addition to the reduced antioxidant defense, can accelerate the progression of HCC. To be more specific, oxidative stress damage affects the gene expression of cellular survival, the products of which can promote the proliferation and differentiation of normal cells and eventually lead to the reduction of cellular apoptosis or even the formation of the tumor cells [3].

A great body of studies have disclosed that the tumorigenesis and progression of HCC are implicated with the mutation and abnormal expression and of genes, involving epidermal growth factor receptor (EGFR) [4], cyclin D1 (CCND1) [5], FoxQ1 [6], c-myc [7], as well as mutations of some tumor-suppressor genes. However, in clinic, the serum detection of alpha-fetoprotein (AFP), magnetic resonance imaging (MRI), or dynamic computed tomography (CT) scan are the conventional methods for the diagnosis and treatment of HCC. Lacking the specificity of auxiliary examination biomarker, it was thus difficult for physicians to achieve accurate diagnosis and treatment of HCC as early as possible, so some patients missed the optimal chance for surgery, thus increasing the risk of death [8]. Hence, the identification of specific and sensitive biomarkers which can assistant us to confirm patients at a lower or higher risk of death from HCC is of great significance, not only for more precise diagnosis, optimal treatment, and better prognosis, but also for a comprehensive understanding of the cellular and molecular mechanisms involved in carcinogenesis.

The recent adoption of high-throughput gene microarray in analyzing tumors and normal samples from patients and healthy individuals enables us to share and explore the global molecular landscapes of tumors at multiple levels ranging from somatic mutations and copy number alterations at the genome level to gene expression at transcriptome level, as well as epigenetic alterations [9–11]. However, the application of microarrays in clinic is limited to a great extent because of countless genes identified by gene profiling, lack of both independent validation and repeatability, as well as the complicated statistical analyses. To put these expression profiles in clinical practice as quickly as possible, it is necessary to identify a suitable amount of genes and develop a proper approach that could be operated by routine assay.

In this study, we downloaded GSE41804 from the Gene Expression Omnibus (GEO, http://www.ncbi.nlm.nih.gov/geo/) and utilized the GEO2R online tool to comprehensively identify the differentially expressed genes (DEGs). Whereafter, we established protein-protein interaction (PPI) network of the DEGs and selected the top 15 hub genes with a high degree of connectivity. Furthermore, we analyzed the gene ontology involving biological process (BP), molecular function (MF), cellular component (CC), and KEGG pathways of the DEGs. Additionally, we constructed three modules and verified their enriched pathways. Meanwhile, overall survival (OS) analysis of the top 15 hub genes were carried out based on the Gene Expression Profiling Interactive Analysis online database (http://gepia.cancer-pku.cn/). Finally, we chose 3 genes to further identify the correlation by comparing the level of the 3 genes and their protein expression in tumor and normal tissues based on The Human Protein Atlas database (http://www.proteinatlas.org).

## 2. Materials and Methods

### 2.1. Microarray Data

We downloaded the gene expression profile of GSE41804 from the GEO database, which was a free and publicly available database. The GSE41804 dataset has a total of 40 samples, containing 20 HCC samples and 20 normal liver tissues, which was based on agilent GPL570 platform ([HG-U133_Plus_2] Affymetrix Human Genome U133 Plus 2.0 Array) by Hodo et al. We also downloaded the Series Matrix File of GSE41804 from the GEO database.

### 2.2. Screen Genes of Differential Expression

The differentially expressed genes (DEGs) between HCC samples and normal liver samples were analyzed using GEO2R (https://www.ncbi.nlm.nih.gov/geo/geo2r/), an interactive online analysis tool for the GEO database, which was based on R language. We defined DEGs as differentially expressed with logFC < −2 (upregulated genes) or logFC > 2 (downregulated genes), according to the criteria described in [12, 13]. The adjusted *P* value < 0.05 was regarded statistically significant, which was used to decrease the false positive rate. Then, 503 DEGs were found, including 360 upregulated genes and 143 downregulated genes, and we selected the top 15 genes with a high degree of connectivity as hub genes.

In addition, we used visual hierarchical cluster analysis to show the heat map and volcano plot of two groups by ImageGP (http://www.ehbio.com/ImageGP/index.php/Home/Index/index.html) after the relative raw data of TXT files were downloaded.

### 2.3. Gene Ontology and KEGG Pathway Analysis of DEGs

Gene ontology (GO) analysis can annotate genes and their products with functions involving cellular components, molecular function, as well as biological pathways [14]. The Kyoto Encyclopedia of Genes and Genomes (KEGG) is a collection of databases that could handle genomes and biological pathways associated with diseases and drugs. KEGG essentially is a resource for the comprehensive understanding of biological systems and some high-level genome functional information [15]. The Database for Annotation, Visualization and Integrated Discovery (DAVID, http://david.ncifcrf.gov) (version 6.7) is an online biological information database which has integrated a bulk of biological data and corresponding analysis tools, thereby providing systematic and comprehensive biological function annotation information for high throughput gene expression [16]. *P* < 0.05 was regarded as the cut-off criterion with statistic difference. To visualize the key molecular functions, biological processes, cellular components, as well as pathways of DEGs, biological analyses were carried out by the DAVID online database.

### 2.4. PPI Network and Module Analysis

The Search Tool for the Retrieval of Interacting Genes (STRING) is an online tool that was designed to assess and integrate the protein-protein interaction (PPI) information, such as physical and functional associations. Up to now, a total of 9,643,763 proteins from 2031 organisms have been covered in STRING version 10.0 [17] To evaluate the interactional correlation of these DEGs, we first drew DEGs by STRING and then utilized the Cytoscape software to construct a PPI network. Meanwhile, we set a maximum number of interactors = 0 and a confidence score ≥ 0.4 as the cut off criterion. Additionally, the Molecular Complex Detection (MCODE) app was also employed to select modules of the PPI network in the Cytoscape according to node score cut − off = 0.2, degree cut − off = 2, max. depth = 100, and *k* − core = 2. The pathway analysis of genes in the three modules was carried out based on DAVID, respectively. Also, 15 hub genes were mapped into STRING according to confidence score ≥ 0.4 and maximum number of interactors ≤ 5. We also used GO and KEGG pathway analysis to investigate their potential information.

### 2.5. Comparison of the Hub Gene Expression Level

GEPIA (http://gepia.cancer-pku.cn/index.html) is a newly developed interactive web server designed by Zefang Tang, Chenwei Li, and Boxi Kang of Zhang Lab, Peking University, aimed at analyzing the RNA sequencing expression data of 9736 tumors and 8587 normal samples from the TCGA and the GTEx projects, using a standard processing pipeline. GEPIA provides customizable functions such as tumor/normal differential expression analysis, profiling according to cancer types or pathological stages, patient survival analysis, similar gene detection, correlation analysis, and dimensionality reduction analysis [18]. In our study, we mainly employed the boxplot to visualize the expression of hub genes in HCC and normal liver tissues. Then we selected two suspicious genes to analyze their correlation in a scatter diagram. The Human Protein Atlas (HPA, https://www.proteinatlas.org/) is a Swedish-based program initiated in 2003 with the aim to map all human proteins in cells, tissues, and organs using the integration of various omics technologies, including antibody-based imaging, mass spectrometry-based proteomics, transcriptomics, and systems biology [19]. By acquiring immunohistochemical data of patients with or without HCC based on HPA, we further verified the expression of these hub genes.

### 2.6. Survival Analysis of Hub Genes

The relapse-free and overall survival information were based on GEPIA database. The hazard ratio (HR) with 95% confidence intervals and logrank *P* value were calculated and displayed on the plot. *P* < 0.05 was considered statistically significant.

### 2.7. Gene Set Enrichment Analysis

20 HCC samples from GSE41804 were divided into two groups (high versus low) according to the expression level of CCNB2, and the median expression value was regarded as the cut-off point. In order to investigate the potential function of CCNB2, GSEA (http://software.broadinstitute.org/gsea/index.jsp) was carried out between the two groups. Annotated gene sets c2.cp.kegg. v5.2.symbols.gmt, sets c2.cp.bp. v5.2.symbols.gmt, sets c2.cp.mf. v5.2.symbols.gmt, and sets c2.cp.cc. v5.2.symbols.gmt were selected as the reference gene sets. FDR < 0.05, |enrichment score (ES)∣ > 0.5 and gene size ≥ 100 were regarded as the cut-off criteria.

### 2.8. Reidentification of Oxidative Stress in HCC

To further verify the vital role of oxidative stress in HCC, we detected the level of some typical markers in carcinoma tissue and adjacent tissue obtained from 6 patients with HCC. The level of superoxide dismutase (SOD), malondialdehyde (MDA), and glutathione peroxidase (GSH-Px) were determined using an assay kit (Beyotime, China) according to the standard operational process. Meanwhile, the mRNA level of NADPH P67 and gp91 were also measured in carcinoma tissue and adjacent tissue. Additionally, the protein expression of SOD, 4-hydroxynonenal (4-HNE), and p65 were also quantified using Western blot. Immunohistochemical staining further verified the expression of 4-HNE in carcinoma tissue and adjacent tissue. The study was carried out in accordance with legal requirements and supported by the Ethics Committee of Renmin Hospital of Wuhan University.

### 2.9. Statistical Analysis

All values are presented as the mean ± SD. All statistical analyses were performed by SPSS 19.0 software. A difference of *P* < 0.05 was considered statistically significant.

## 3. Results

### 3.1. Identification of DEGs and Hub Genes

**Figure 1:**
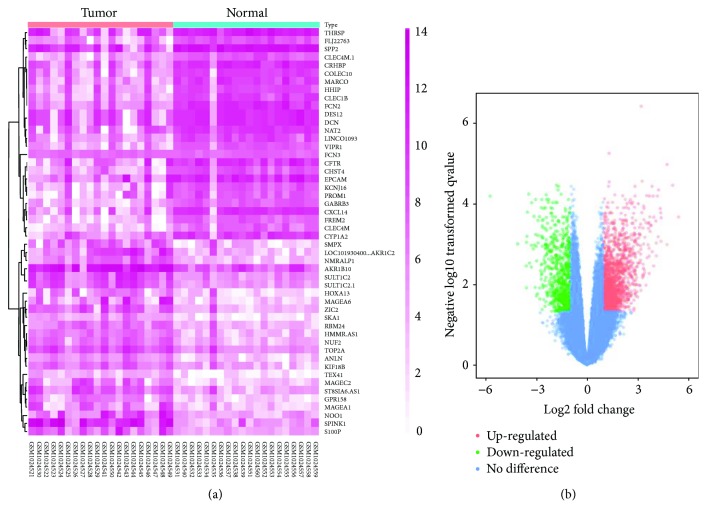
(a) Heat map of 50 representative DEGs. (b) Volcano plot of genes detected in HCC. Red means upregulated DEGs; green means downregulated DEGs; blue means no difference.

There were 20 HCC samples and 20 normal samples in the study. The GEO2R online analysis tool was applied to detect the DEGs, using adjusted *P* value < 0.05 and logFC ≥ 2 or logFC ≤ −2 as cut-off criteria. A total of 503 DEGs were captured after analyzing GSE41804, 360 of which were downregulated genes while 143 were upregulated (Figure 1(b)). The expression level of the top 50 DEGs with fold change 2 was displayed in (Figure 1(a)). Additionally, 15 hub genes were identified according to their degree of connectivity from high to low (Table1).

### 3.2. GO Function and KEGG Pathway Enrichment Analysis

In order to obtain a more comprehensive and in-depth knowledge of those chosen DEGs, GO function and KEGG pathway enrichment analysis were employed via DAVID. After importing all the DEGs to the DAVID software, we discovered upregulated DEGs and downregulated DEGs by GO analysis. To be more specific, these DEGs were mainly enriched in biological processes (BP), involving epoxygenase P450 pathway; oxidation-reduction process; cellular response to zinc ion; negative regulation of growth; and exogenous drug catabolic process for downregulation, mitotic nuclear division, cell division, sister chromatid cohesion, chromosome segregation, and protein localization to kinetochore for upregulation. As for function (MF), the downregulated DEGs were mainly implicated with oxidoreductase activity (acting on paired donors, with incorporation or reduction of molecular oxygen), oxygen binding, iron ion binding, monooxygenase activity, and oxidoreductase activity (acting on paired donors, with incorporation or reduction of molecular oxygen, reduced flavin or flavoprotein as one donor, and incorporation of one atom of oxygen). The upregulated DEGs were mainly responsible for protein binding, microtubule motor activity, microtubule binding, chromatin binding, and cyclin-dependent protein serine/threonine kinase activity. In addition, GO cell component (CC) analysis uncovered that the downregulated DEGs were principally enriched in the extracellular region, extracellular space, organelle membrane, integral component of plasma membrane, and basolateral plasma membrane, while the upregulated DEGs were mainly enriched in midbody, kinetochore, condensed chromosome kinetochore, chromosome, centromeric region, as well as kinesin complex (Table 2).

**Figure 2:**
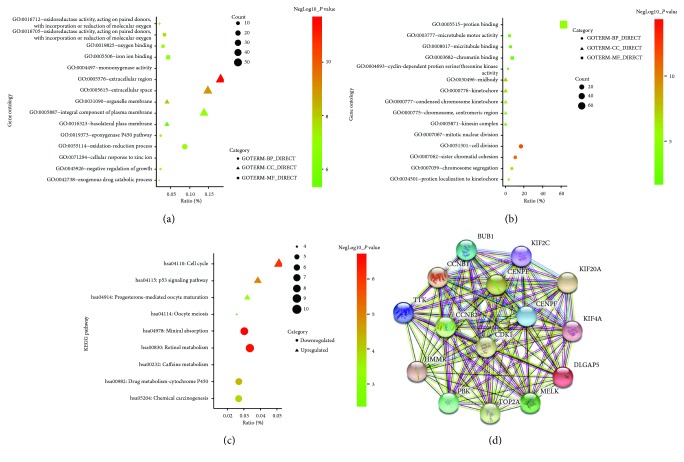
(a) GO analysis of downregulated DEGs. (b) GO analysis of upregulated DEGs. (c) KEGG pathway of DEGs. (d) The protein-protein interaction (PPI) network of the top 15 hub genes.

Table 3 displayed the most significantly enriched KEGG pathway of the upregulated and downregulated DEGs. These downregulated DEGs were enriched in mineral absorption, retinol metabolism, caffeine metabolism, drug metabolism-Cytochrome P450, and chemical carcinogenesis, while the upregulated DEGs were enriched in cell cycle, p53 signaling pathway, progesterone-mediated oocyte maturation, and oocyte meiosis. Figures 2(a)–2(c) gives a GO and KEGG pathway enrichment plot of HCC.

### 3.3. Hub Genes and Module Screening from the PPI Network

Based on the information of the STRING protein query from public databases, we constructed the PPI network of the top 15 hub genes according to the degree of connectivity (Figure 2(d)). The top 15 hub genes with a higher degree of connectivity are as follows: TOP2A, CDK1, CCNB1, BUB1, CENPF, CCNB2, TTK, KIF2C, HMMR, MELK,CENPE, KIF20A, KIF4A, PBK, and DLGAP5. By the Kaplan-Meier plotter, we found that a total of 14 hub genes contributed to worse overall survival situation except CCNB2. Again, based on the GO function, KEGG pathway analysis, and the survival analysis, we unveiled that CCNB1, CDK1, BUB1, and TTK were enriched in cell cycle.

**Figure 3:**
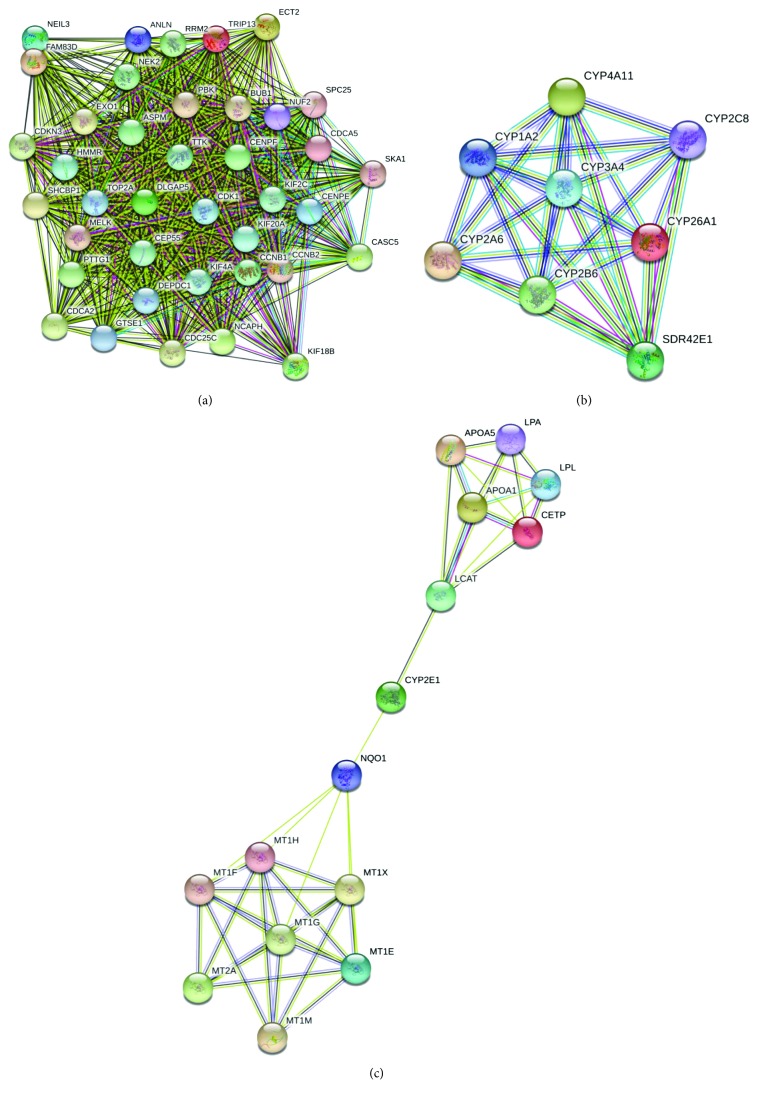
Top 3 modules from the protein-protein interaction network: (a) module 1, (b) module 2, and (c) module 3.

In order to detect the most significant modules in this PPI network, we employed the MCODE plug-in. The top 3 modules were selected (Figure 3). KEGG pathway analysis disclosed that the top 3 modules were mainly associated with cell cycle, oxidative stress, and liver detoxification (Table 4).

### 3.4. The Kaplan-Meier Plotter and Expression Level of Hub Genes

**Figure 4:**
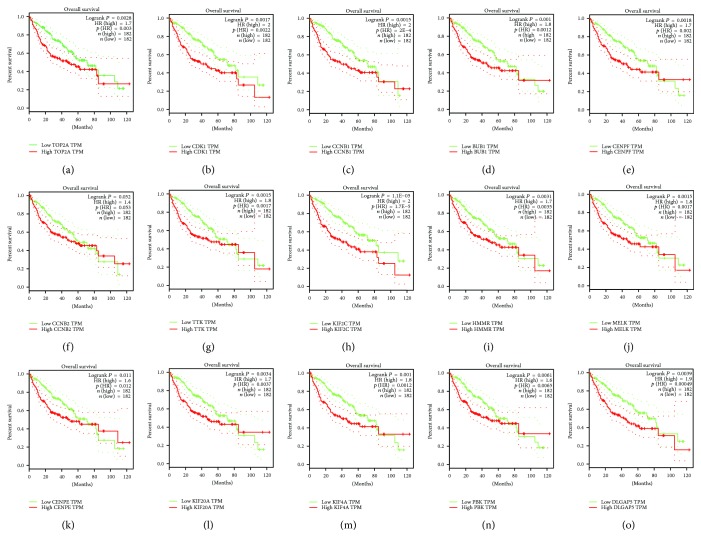
Prognostic value of 15 genes ((a) TOP2A, (b) CDK1, (c) CCNB1, (d) BUB1, (e) CENPF, (f) CCNB2, (g) TTK, (h) KIF2C, (i) HMMR, (j) MELK, (k) CENPE, (l) KIF20A, (m) KIF4A, (n) PBK, and (o) DLGAP5) in HCC. *P* < 0.05 was regarded statistically different.

We obtained the prognostic information of the top 15 hub genes in http://gepia.cancer-pku.cn/. It was demonstrated that expression of TOP2A (HR (high) = 0.003, logrank *P* = 0.0028) was associated with worse overall survival (OS) for HCC patients, as well as CDK1(HR (high) = 0.00022, logrank *P* = 0.00017), CCNB1 (HR (high) = 2E − 04, logrank *P* = 0.00015), BUB1 (HR (high) = 0.0012, logrank *P* = 0.001), CENPF (HR (high) = 0.0018, logrank *P* = 0.002), TTK (HR (high) = 0.015, logrank *P* = 0.0017), KIF2C (HR (high) = 1.1*E* − 05, logrank *P* = 1.7*E* − 05), HMMR (HR (high) = 0.0031, logrank *P* = 0.0035), MELK(HR (high) = 0.0015, logrank *P* = 0.0017), CENPE(HR (high) = 0.011, logrank *P* = 0.012), KIF20A (HR (high) = 0.0034, logrank *P* = 0.037), KIF4A (HR (high) = 0.001, logrank *P* = 0.0012), and DLGAP5(HR (high) = 0.00039, Logrank *P* = 0.00049). Only the level of CCNB2 (HR (high) = 0.053, logrank *P* = 0.052) had no obvious difference on the survival curve of HCC patients (Figure 4).

**Figure 5:**
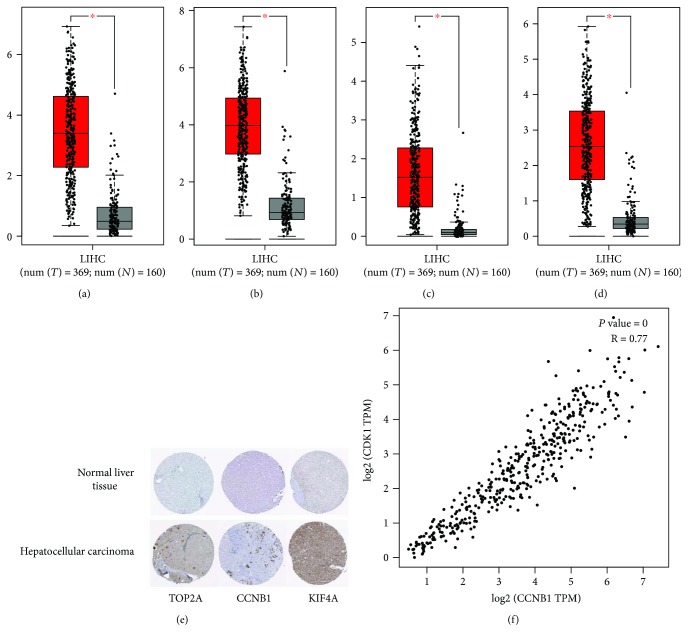
(a–d) Expression level of TOP2A, CCNB1, KIF4A, and CCNB2 in HCC and normal tissues. Number (*T* = 369, *N* = 160); ^∗^*P* < 0.05. (e) TOP2A, CCNB1, and KIF4A protein were strongly upregulated in HCC tissues compared with normal liver tissues based on The Human Protein Atlas database. The normal liver tissue of TOP2A was from a female, aged 32, (patient ID: 1846; staining: not detected; intensity: negative; quantity: negative; location: none), and the HCC tissue was from a male, aged 80 (patient ID: 2280; staining: low; intensity: moderate; quantity: <25%; location: cytoplasmic/membranous nuclear). The normal liver tissue of CCNB1 was from a female, ages 63, (patient ID: 3222; staining: not detected; intensity: negative; quantity: negative; location: none), and the HCC tissue was from a female, aged 41, (patient ID: 5037; staining: moderate; intensity: weak; quantity: <25%; location: cytoplasmic/membranous nuclear). The normal liver tissue of KIF4A was from a female, aged 54 (patient ID: 3402; staining: not detected; intensity: negative; quantity: negative; location: none), and the HCC tissue was from a male, aged 67, (patient ID: 3477; staining: moderate; intensity: moderate; quantity: >75%; location: cytoplasmic/membranous nuclear). (f) The correlation analysis between CCNB1 and CDK1. CCNB1 and CDK1 are obviously positively correlated.

Then, we selected 4 hub genes based on the KEGG pathways (Table 5) of the top 15 genes to verify the expression level in liver tissues between HCC and healthy people using GEPIA, and Figures 5(a)–5(d) showed that compared to normal group, the expression level of TOP2A, CCNB1, KIF4A, and CCNB2 significantly elevated in HCC patients. Intriguingly, CCNB2 in fact had no influence on the prognosis of HCC patients. Subsequently, we searched the immunohistochemical data in the HPA website. (The protein expression of CCNB2 was absent in HPA.) The staining pictures demonstrated that the TOP2A, CCNB1, and KIF4A in HCC patients exhibited higher expression levels compared with those in healthy individuals, which further verified the results of boxplots from GEPIA (Figure 5(e)).

Finally, we selected two most suspicious genes (CCNB1 and CDK1) based the KEGG analysis of the top 15 hub genes. CCNB1 and CDK1 were two genes which were implicated with cell cycle, progesterone-mediated oocyte maturation, and p53 signaling pathway (Table 5). Using the correlation analysis in GEPIA, we found that CCNB1 and CDK1 are obviously positively correlated (*P* value = 0, *R* = 0.77) (Figure 5(f)).

### 3.5. Gene Set Enrichment Analysis

**Figure 6:**
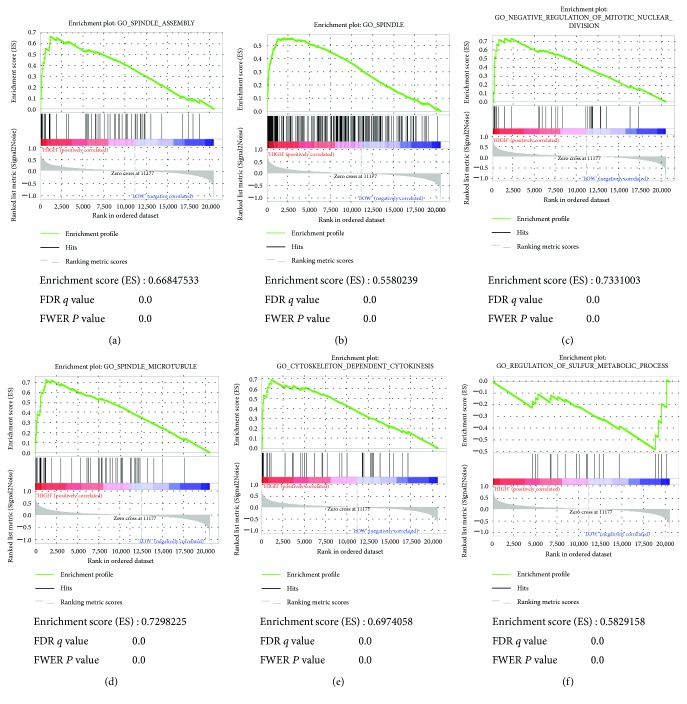
Gene set enrichment analysis (GSEA). Listed pictures are 10 representative functional gene sets enriched in HCC with CCNB2 highly expressed.

To acquire further insight into the function of the hub gene, GSEA was conducted to map into GO analysis and KEGG pathways database. Under the cut-off criteria FDR < 0.05, |enrichment score (ES)∣ > 0.6, and gene size ≥ 100, a total of 6 functional gene sets were enriched, which mainly focused on pathways associated with cell proliferation and differentiation. The six pathways were “spindle assembly,” “spindle,” “negative regulation of mitotic nuclear division,” “spindle microtubule,” “cytoskeleton dependent cytokinesis,” and “regulation of sulfur metabolic process” (Figure 6).

### 3.6. Oxidative Stress Is Activated in HCC

**Figure 7:**
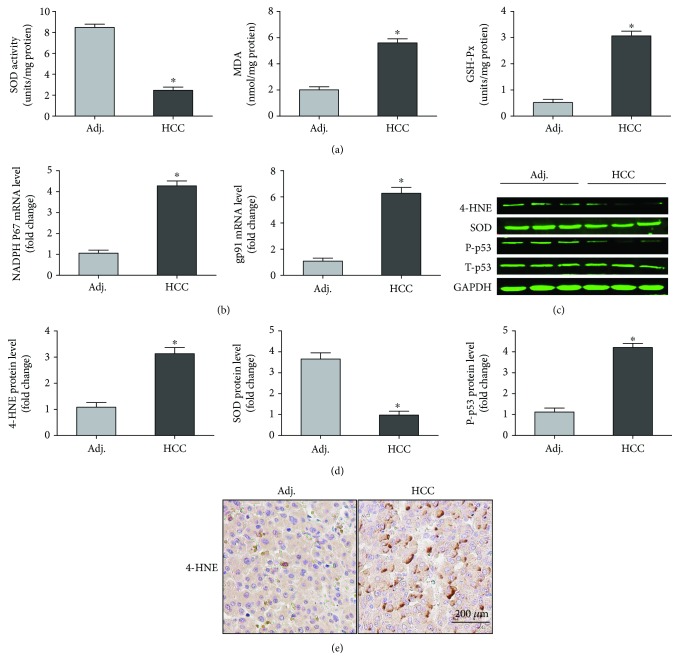
Reidentification of oxidative stress in HCC. (a) SOD activity, MDA production, and GSH-Px activity in the indicated group (*n* = 6). (b) The mRNA levels of NADPH P67 and gp91. (*n* = 6). (c, d) Western blotting analysis and quantitative data of oxidative stress (*n* = 6). (e) Representative images of immunohistochemical staining for the 4-HNE protein. ^∗^*P* < 0.05 versus Adj. group. Adj: adjacent tissue; HCC: hepatocellular carcinoma.

Consistent with what we have predicted, the level of oxidative stress was significantly enhanced in carcinoma tissue compared with adjacent tissue. In detail, the activity of SOD, one of the typical antioxidant enzymes, was decreased in carcinoma tissue while the activity of GSH-Px as well as the level of MDA were increased in carcinoma tissue (Figure 7(a)). Meanwhile, the mRNA level of NADPH P67 and gp91 were also significantly upregulated in the HCC tissue (Figure 7(b)). The results from the Western blot and immunohistochemical staining further identified the protein level of these biomarkers associated with oxidative stress (Figures 7(c)–7(e)). Taken together, our experiments demonstrated that oxidative stress was activated in HCC, which further proved our hypothesis from bioinformatics.

## 4. Discussion

Trends of HCC mortality rates have elevated over recent decades worldwide. Although the diagnostic and treatment approaches have developed a lot recently, the prognosis of HCC is still poor [20]. Thus, specific and sensitive biomarkers for HCC are urgently needed to be selected. High-throughput research can facilitate the in-depth exploration of the vital mechanisms contributing to HCC. Our study systematically focused on expression profiling obtained from microarray studies of HCC. Our analysis included 20 HCC samples and 20 normal samples from the GEO database of GSE41804. A total of 503 DEGs were captured involving 360 upregulated genes and 143 downregulated genes. To have a better exploration of these DEGs, we carried out GO function and KEGG pathway analysis of these DEGs.

### 4.1. Oxidative Stress Is Critical in the Carcinogenesis of HCC

Although many mechanisms have been disclosed to contribute to the progression of HCC, the predominant mechanism implicated with tumorigenesis is still controversial, which brings some difficulties to the diagnosis and treatment of HCC. Gene ontology and PPI analysis in our study showed that downregulated DEGs were primarily involved in oxidative stress. Our experimental results in carcinoma tissue and adjacent tissue further identified the vital role of oxidative stress in HCC.

To our knowledge, oxidative stress is recognized to play a vital part in the initiation and promotion of carcinogenesis because it can occur and overproduce ROS and RNS through endogenous or exogenous insults [21]. On the one hand, for the reason that polymorphonuclear neutrophils (PMNs) are a major source of ROS in an inflamed liver, oxidative stress acts as a core player in the pathogenesis of chronic liver diseases and precancerous lesions infected by hepatitis B virus (HBV) or hepatitis C virus (HCV) [22]. On the other one hand, nonparenchymal cells including macrophages and Kupffer cells, which can release cytokines, are another incentive of ROS production in hepatocytes [23]. To be more specific, prolonged or upregulated ROS production is associated with modification and mutation of gene expression in HCC. Particularly, unrepaired damage induced by oxidative stress to DNA could give rise to mutations, given that the repair of modified bases ensues later than cell replication. Apart from oxidative nuclear DNA damage, formation of mitochondrial DNA damage or mutation as well as alteration of mitochondrial genomic function have also been unveiled to induce the occurrence of carcinogenesis [3, 24]. Thus, the level of oxidative stress can be a promising predictor in the diagnosis and treatment of HCC.

Additionally, our gene ontology analysis indicated that the downregulated DEGs were primarily related with the alteration of extracellular structure including extracellular space, organelle membrane, integral component of plasma membrane, and basolateral plasma membrane. Increased extracellular matrix remodeling has also been proved to be associated with tumor progression in human HCC. For instance, matrix metalloproteinase-2 (MMP-2) is an important enzyme in the process of extracellular matrix remodeling implicated with tumor invasion and metastasis. Overexpression of MMP2 seemed strikingly associated with HCC because MMP2 in the tumor is mainly responsible for the fibrogenesis [25]. As reported, hepatic stellate cells, acting as main connective tissue cells in the liver, could be activated by oxidative stress and then produce extracellular matrix which is essential for normal growth and differentiation of cells after liver damage [26]. Our result suggests that oxidative stress in HCC not only directly affects the progression of HCC, but also interacts with other events to modulate HCC.

What is more, KEGG analysis shows that the upregulated DEGs are implicated with p53 signaling. Our data from Western blot also identified that the expression of P-p53 in HCC tissue was significantly higher than that in adjacent tissue. To our knowledge, p53 genes are one of the sensitive redox transcription factor which could be upregulated by enhancing its translational speed and posttranslational modification when the DNA is damaged by oxidative stress in cells. The posttranslational modification of oxidative stress in cell, such as phosphorylation, ubiquitination, sumoylation, acetylation, and methylation, may cause conformational and locational changes of p53 and then affect its downstream targets [27, 28]. Taken together, strategies to suppress the alteration of oxidative stress or regulate various posttranscriptional modifications of p53 and formation of extracellular matrix through oxidative stress are helpful to develop drugs for the treatment of HCC.

### 4.2. Targeting Cell Cycle Could Be a Potential Strategy for Therapy of HCC

Dysregulated cell cycle-mediated cell transformation and uncontrolled cell growth are some of the fundamental biological features of malignant tumors. Amplification of cyclin genes, especially cyclins D and E, is a crucial event process which occurs in the HCC [29, 30]. Masaki et al. found that in Long-Evans Cinnamon (LEC) rats, with the progression of HCC, cyclin D1-related kinase activities were dramatically enhanced, in particular, the cyclin D1-related enzymatic activity. On the contrary, the activity was relatively low in the 2-month-old LEC rats as well as in the control rats [31]. The top 15 hub genes with a higher degree of connectivity in our analysis demonstrated the significance of cell cycle in the progression of HCC. Most of these hub genes including CDK1 [32], CENPF [33], CCNB2 [34], MELK [35], CENPE [36], KIF20A [37], KIF4A [38], PBK [39], and DLGAP5 [40] have been proven to be responsible for the cell cycle-associated proliferation and differentiation of tumor. Additionally, GO analysis of DEGs showed that all upregulated DEGs were also involved in cell cycle including mitotic nuclear division, cell division, sister chromatid cohesion, midbody, protein localization to kinetochore, and microtubule binding, which indicated that the altered expression of vital genes in HCC should be associated with cell cycle. KEGG pathway analysis further verified the hypothesis with the downregulated DEGs associated with cell cycle and p53 signaling pathway. Taken together, from our perspective based on the GO and KEGG analysis, drugs targeting the cell cycle of cancer cells may be a potential strategy for therapy of HCC.

### 4.3. Altered Cell Cycle, Dysfunction of Cytochrome P450, and Impaired Liver Detoxification Effect Are 3 Typical Modules in PPI Network for HCC

PPI is defined as the process by which two or more kinds of protein molecules form a protein complex by noncovalent bonding. The PPI network could provide a visible framework for a better understanding of the functional organization of the proteome [41]. From the enriched pathways of top 3 modules, we uncovered that the interactions among the proteins in HCC mainly concentrated on pathways implicated with cell cycle, Cytochrome P450, and liver detoxification.

Cytochrome P450 is a big family of enzymes localizing to either the endoplasmic reticulum or mitochondrial membranes, which exert various important roles in the process of metabolizing endogenous and exogenous molecules, especially some drugs [42]. Previous studies have shown that Cytochrome P450 genetic polymorphisms exhibited a certain association with the risk of HCC in patients carrying chronic hepatitis B [43]. In HCC patients, the levels of some Cytochrome P450 also changed with the progression of HCC. For instance, CYP2J2, a member in Cytochrome P450 family, was discovered to have critical roles in the proliferation and resistance to the anticancer drug (doxorubicin) in HepG2 cells by decreasing the ratio of Bax/Bcl622 ratio and elevating pro62caspase623 levels [44]. Genetic polymorphisms of some Cytochrome P450 enzymes, such as CYP2D6^∗^10, have been proven to have influence on enzyme activity. In HCC patients, CYP2D6^∗^10 allelic frequency was obviously different compared with the control individuals [45]. Consistent with these findings, module 2 showed that the PPI in tumor groups and normal groups involved many members in Cytochrome P450, such as CYP3A4, CYP4A11, CYP2B6, CYP2C8, CYP26A1, CYP2A6, and CYP1A2, indicating that some key Cytochrome P450 enzymes had essential functions in HCC.

In addition, metallothionein is also involved in the differentiation and proliferation of tumor cells. The relationship between metallothionein and tumors mainly focused on the metallothionein and tumorigenesis, toxic side effects of ant-tumor drugs, as well as drug resistance. Metallothionein expression defect is one of the symptoms of cancer; therefore, the in-depth study of the relationship between metallothionein and tumors is expected to obtain the target drugs for the treatment of cancer [46]. Metallothionein is expressed at a high level in liver tissues, which is mainly responsible for liver detoxification and can be significantly induced by a variety of drugs [47]. Datta et al. [48] demonstrated that in hepatocarcinogenesis, the expression of metallothionein was significantly blocked through inactivation of CCAAT/enhancer-binding protein *α* by phosphatidylinositol 3-kinase (PI3K) signaling pathway, which eventually caused poor liver detoxification. Base on module 3 from the PPI network, we found that the network mainly concentrated on metallothionein, suggesting the vital significance of metallothionein from the view of bioinformatics. Hence, monitoring some indexes associated with metallothionein is of great importance for the diagnosis and treatment of HCC.

### 4.4. CCNB2 May Be One of the Switches of HCC but Cannot Promote Tumor Progression

As the Kaplan-Meier plotter showed, the survival curve displayed no significant difference in HCC patients with low expression and high expression of CCNB2. Intriguingly, in the HCC group and healthy group, the expression of CCNB2 exhibited obvious difference in the box diagram.

To our knowledge, CCNB2 (Cyclin B2), which belelongs to one of the members in cyclin family proteins, has a core role in G2/M transition of tumor. CCNB2 has been found to be upregulated in many types of human tumors [49]. In Chinese non-small-cell lung cancer (NSCLC) patients, high expression levels of CCNB2 protein were positively correlated with the tumor size, status of differentiated degree, distant metastasis, lymph node metastasis, as well as clinical stage [50]. However, expression levels of CCNB2 do nothing with survival condition in HCC patients, while the levels of CCNB2 in HCC patients have obvious difference compared to the health group, which indicates that CCNB2 may act as a switch to initiate the occurrence of HCC, but it does not exert other tumor-related effects after the tumorigenesis. In fact, in the primary HCC tissue samples, CCNB2 has been unveiled to be regulated by its upstream karyopherin subunit-*α* 2, which can inhibit cell proliferation and induce cell cycle arrest in the G2/M phase [34]. Based on GSEA, we also found that the gene sets associated with cell cycle-related pathways were enriched in the samples with FCER1G highly expressed HCC patients. But further study is required to clarify the process of CCNB2 transcriptional activation, the effect of CCNB2 on cell cycle progression, and how this affects initiation of HCC.

In conclusion, we offer a novel and comprehensive analysis of gene expression profiles to identify DEGs, which may play core roles in the occurrence, development, and prognosis in patients with HCC. Genes involved in oxidative stress and cell cycle, Cytochrome P450, and metallothionein were significantly changed in HCC patients. To obtain more accurate correlation results, we intend to initiate subsequent identification experiments later to verify these predictive results. Taken together, we sincerely hope that this analysis will offer valuable and powerful information for future research on the molecular mechanisms contributing to HCC and provide clues for the discovery of novel diagnosis biomarkers and therapeutic strategies.

## Figures and Tables

**Table 1:** Top 15 hub genes with higher degree of connectivity.

Gene	Degree of connectivity	Adjusted *P* value
TOP2A	48	2.13*E* − 04
CDK1	43	1.87*E* − 03
CCNB1	42	3.57*E* − 04
BUB1	42	1.31*E* − 03
CENPF	41	2.23*E* − 04
CCNB2	40	3.11*E* − 04
TTK	40	1.06*E* − 03
KIF2C	40	5.80*E* − 03
HMMR	40	2.10*E* − 04
MELK	40	9.21*E* − 04
CENPE	39	2.49*E* − 03
KIF20A	39	1.33*E* − 04
KIF4A	39	4.66*E* − 04
PBK	39	1.07*E* − 03
DLGAP5	39	1.01*E* − 03

**Table 2:** Gene ontology analysis of differentially expressed genes associated with hepatocellular carcinoma.

Expression	Category	Term	Count	%	*P* value	FDR
Downregulated	GOTERM_BP_DIRECT	GO:0019373~epoxygenase P450 pathway	7	0.02	5.20*E* − 08	8.49*E* − 05
	GOTERM_BP_DIRECT	GO:0055114~oxidation-reduction process	26	0.09	7.31*E* − 08	1.19*E* − 04
	GOTERM_BP_DIRECT	GO:0071294~cellular response to zinc ion	7	0.02	7.53*E* − 08	1.23*E* − 04
	GOTERM_BP_DIRECT	GO:0045926~negative regulation of growth	7	0.02	7.53*E* − 08	1.23*E* − 04
	GOTERM_BP_DIRECT	GO:0042738~exogenous drug catabolic process	6	0.02	1.95*E* − 07	3.19*E* − 04
	GOTERM_CC_DIRECT	GO:0005576~extracellular region	54	0.18	2.46*E* − 12	3.08*E* − 09
	GOTERM_CC_DIRECT	GO:0005615~extracellular space	44	0.14	1.19*E* − 09	1.49*E* − 06
	GOTERM_CC_DIRECT	GO:0031090~organelle membrane	12	0.04	5.53*E* − 09	6.91*E* − 06
	GOTERM_CC_DIRECT	GO:0005887~integral component of plasma membrane	41	0.14	1.42*E* − 07	1.77*E* − 04
	GOTERM_CC_DIRECT	GO:0016323~basolateral plasma membrane	12	0.04	9.02*E* − 06	0.01
	GOTERM_MF_DIRECT	GO:0016705~oxidoreductase activity, acting on paired donors, with incorporation or reduction of molecular oxygen	10	0.03	2.41*E* − 08	3.42*E* − 05
	GOTERM_MF_DIRECT	GO:0019825~oxygen binding	9	0.03	8.01*E* − 08	1.14*E* − 04
	GOTERM_MF_DIRECT	GO:0005506~iron ion binding	13	0.04	3.31*E* − 07	4.70*E* − 04
	GOTERM_MF_DIRECT	GO:0004497~monooxygenase activity	9	0.03	4.36*E* − 07	6.19*E* − 04
	GOTERM_MF_DIRECT	GO:0016712~oxidoreductase activity, acting on paired donors, with incorporation or reduction of molecular oxygen, reduced flavin or flavoprotein as one donor, and incorporation of one atom of oxygen	6	0.02	6.52*E* − 07	9.26*E* − 04

Upregulated	GOTERM_BP_DIRECT	GO:0007067~mitotic nuclear division	19	16.67	5.25*E* − 15	7.80*E* − 12
	GOTERM_BP_DIRECT	GO:0051301~cell division	19	16.67	1.94*E* − 12	2.90*E* − 09
	GOTERM_BP_DIRECT	GO:0007062~sister chromatid cohesion	12	10.53	2.16*E* − 11	3.23*E* − 08
	GOTERM_BP_DIRECT	GO:0007059~chromosome segregation	8	7.02	1.44*E* − 07	2.16*E* − 04
	GOTERM_BP_DIRECT	GO:0034501~protein localization to kinetochore	4	3.51	2.31*E* − 05	0.03
	GOTERM_CC_DIRECT	GO:0030496~midbody	10	0.06	2.85*E* − 08	3.40*E* − 05
	GOTERM_CC_DIRECT	GO:0000776~kinetochore	8	0.05	2.45*E* − 07	2.93*E* − 04
	GOTERM_CC_DIRECT	GO:0000777~condensed chromosome kinetochore	7	0.04	6.94*E* − 06	0.01
	GOTERM_CC_DIRECT	GO:0000775~chromosome, centromeric region	6	0.03	1.29*E* − 05	0.02
	GOTERM_CC_DIRECT	GO:0005871~kinesin complex	5	0.03	1.81*E* − 04	0.22
	GOTERM_MF_DIRECT	GO:0005515~protein binding	72	63.16	4.54*E* − 06	0.01
	GOTERM_MF_DIRECT	GO:0003777~microtubule motor activity	5	4.39	0.001073553	1.34
	GOTERM_MF_DIRECT	GO:0008017~microtubule binding	6	5.26	0.006401849	7.77
	GOTERM_MF_DIRECT	GO:0003682~chromatin binding	8	7.02	0.006540341	7.93
	GOTERM_MF_DIRECT	GO:0004693~cyclin-dependent protein serine/threonine kinase activity	3	2.63	0.015639967	18.01

GO: gene ontology; FDR: false discovery rate.

**Table 3:** KEGG pathway analysis of differentially expressed genes associated with hepatocellular carcinoma.

Category	Term	Count	%	*P* value	Genes	FDR
Downregulated DEGs	hsa04978: mineral absorption	9	0.03	2.22*E* − 07	MT1M, SLC5A1, MT2A, MT1E, TRPV6, MT1H, MT1X, MT1G, and MT1F	2.68*E* − 04
	hsa00830: retinol metabolism	10	0.03	2.90*E* − 07	CYP3A4, CYP4A11, CYP2B6, CYP2C8, ADH4, ADH1B, CYP26A1, CYP2A6, CYP1A2, and RDH16	3.50*E* − 04
	hsa00232: caffeine metabolism	4	0.01	2.79*E* − 05	XDH, NAT2, CYP2A6, and CYP1A2	0.03
	hsa00982: drug metabolism-cytochrome P450	8	0.03	4.75*E* − 05	CYP3A4, CYP2B6, CYP2C8, ADH4, ADH1B, CYP2A6, CYP2E1, and CYP1A2	0.06
	hsa05204: chemical carcinogenesis	8	0.03	1.35*E* − 04	CYP3A4, CYP2C8, ADH4, NAT2, ADH1B, CYP2A6, CYP2E1, and CYP1A2	0.16

Upregulated DEGs	hsa04110: cell cycle	8	0.05	9.30*E* − 07	CCNB1, CDK1, CCNB2, BUB1, TTK, PTTG1, SFN, and CDC25C	9.18*E* − 04
	hsa04115: p53 signaling pathway	6	0.04	1.03*E* − 05	CCNB1, CDK1, CCNB2, RRM2, SFN, and GTSE1	0.01
	hsa04914: progesterone-mediated oocyte maturation	5	0.03	5.69*E* − 04	CCNB1, CDK1, CCNB2, BUB1, and CDC25C	0.56
	hsa04114: oocyte meiosis	4	0.03	0.01	CDK1, BUB1, PTTG1, and CDC25C	11.64

KEGG: Kyoto Encyclopedia of Genes and Genomes; FDR: false discovery rate.

**Table 4:** The enriched pathways of top 3 modules.

Module	Term	*P* value	FDR	Genes
Module 1	Cell cycle	2.66*E* − 08	1.96*E* − 05	CCNB1, CDK1, CCNB2, BUB1, TTK, PTTG1, and CDC25C
	ATP binding	3.86*E* − 07	3.59*E* − 04	CDK1, KIF4A, NEK2, KIF18B, TTK, CENPE, PBK, KIF2C, BUB1, TOP2A, MELK, TRIP13, and KIF20A
	Kinesin, motor region, and conserved site	1.54*E* − 06	0.0016579	KIF2C, KIF4A, KIF18B, CENPE, and KIF20A
	Kinesin, motor domain	2.30*E* − 06	0.00247205	KIF2C, KIF4A, KIF18B, CENPE, and KIF20A
	p53 signaling pathway	3.59*E* − 06	0.002649002	CCNB1, CDK1, CCNB2, RRM2, and GTSE1

Module 2	Cytochrome P450, conserved site	4.98*E* − 15	3.11*E* − 12	CYP3A4, CYP4A11, CYP2B6, CYP2C8, CYP26A1, CYP2A6, and CYP1A2
	Cytochrome P450	9.23*E* − 15	5.74*E* − 12	CYP3A4, CYP4A11, CYP2B6, CYP2C8, CYP26A1, CYP2A6, and CYP1A2
	Monooxygenase	3.58*E* − 14	2.84*E* − 11	CYP3A4, CYP4A11, CYP2B6, CYP2C8, CYP26A1, CYP2A6, and CYP1A2
	Organelle membrane	6.92*E* − 14	3.79*E* − 11	CYP3A4, CYP4A11, CYP2B6, CYP2C8, CYP26A1, CYP2A6, and CYP1A2
	Metal ion-binding site: iron (heme axial ligand)	8.64*E* − 14	6.13*E* − 11	CYP3A4, CYP4A11, CYP2B6, CYP2C8, CYP26A1, CYP2A6, and CYP1A2

Module 3	Metal-thiolate cluster	2.15*E* − 17	1.80*E* − 14	MT1M, MT2A, MT1E, MT1H, MT1G, MT1X, MT1F
	Metallothionein, vertebrate, and metal binding site	2.35*E* − 17	1.91*E* − 14	MT1M, MT2A, MT1E, MT1H, MT1G, MT1X, and MT1F
	Metallothionein, vertebrate	5.17*E* − 17	4.19*E* − 14	MT1M, MT2A, MT1E, MT1H, MT1G, MT1X, and MT1F
	Metallothionein domain	5.17*E* − 17	4.19*E* − 14	MT1M, MT2A, MT1E, MT1H, MT1G, MT1X, and MT1F
	Metallothionein superfamily, eukaryotic	5.17*E* − 17	4.19*E* − 14	MT1M, MT2A, MT1E, MT1H, MT1G, MT1X, and MT1F

**Table 5:** KEGG pathway analysis of top 15 hub genes with higher degree of connectivity.

Term	Count	%	*P* value	Genes	FDR
cfa04110: cell cycle	5	0.18	5.08*E* − 07	CCNB1, CDK1, CCNB2, BUB1, and TTK	3.05*E* − 04
cfa04914: progesterone-mediated oocyte maturation	4	0.15	1.93*E* − 05	CCNB1, CDK1, CCNB2, and BUB1	0.01
cfa04115: p53 signaling pathway	3	0.11	8.88*E* − 04	CCNB1, CDK1, and CCNB2	0.53
cfa04114: oocyte meiosis	2	0.07	0.07	CDK1, BUB1	37.98
cfa04068: FoxO signaling pathway	2	0.07	0.09	CCNB1, CCNB2	44.34

KEGG: Kyoto Encyclopedia of Genes and Genomes; FDR: false discovery rate.

## Data Availability

The data used to support the findings of this study are available from the corresponding author upon request.
